# Strategies for Engineering Reproductive Sterility in Plantation Forests

**DOI:** 10.3389/fpls.2018.01671

**Published:** 2018-11-15

**Authors:** Steffi Fritsche, Amy L. Klocko, Agnieszka Boron, Amy M. Brunner, Glenn Thorlby

**Affiliations:** ^1^Scion, Rotorua, New Zealand; ^2^Department of Biology, University of Colorado Colorado Springs, Colorado Springs, CO, United States; ^3^Department of Forest Resources and Environmental Conservation, Virginia Tech, Blacksburg, VA, United States

**Keywords:** sterility, reproduction, forest trees, gene editing, genetic engineering, containment

## Abstract

A considerable body of research exists concerning the development of technologies to engineer sterility in forest trees. The primary driver for this work has been to mitigate concerns arising from gene flow from commercial plantings of genetically engineered (GE) trees to non-GE plantations, or to wild or feral relatives. More recently, there has been interest in the use of sterility technologies as a means to mitigate the global environmental and socio-economic damage caused by the escape of non-native invasive tree species from planted forests. The current sophisticated understanding of the molecular processes underpinning sexual reproduction in angiosperms has facilitated the successful demonstration of a number of control strategies in hardwood tree species, particularly in the model hardwood tree Poplar. Despite gymnosperm softwood trees, such as pines, making up the majority of the global planted forest estate, only pollen sterility, via cell ablation, has been demonstrated in softwoods. Progress has been limited by the lack of an endogenous model system, long timescales required for testing, and key differences between softwood reproductive pathways and those of well characterized angiosperm model systems. The availability of comprehensive genome and transcriptome resources has allowed unprecedented insights into the reproductive processes of both hardwood and softwood tree species. This increased fundamental knowledge together with the implementation of new breeding technologies, such as gene editing, which potentially face a less oppressive regulatory regime, is making the implementation of engineered sterility into commercial forestry a realistic possibility.

## Drivers for Engineering Sterile Forest Trees

Increasing global population coupled with transition to a sustainable bio-based economy is predicted to lead to growing pressure on forests to deliver wood-based products, energy, food, and ecosystem services whilst maintaining their role as major reservoirs of biodiversity. To accommodate this growing demand, it is estimated that the amount of wood we take from forests and plantations each year may need to triple by 2050 (WWF, 2015). Planted forests, which in 2015 made up 7% of forest lands, provide a means to sustainably increase production of forest products and reduce pressure on natural forests (FAO, 2015). Alongside improved silviculture, land management and other technological advances, biotech-based technologies offer tools to enhance the sustainability and productivity of planted forests (Al-Ahmad, 2018). Sterile trees have the ability to impact a number of obstacles to increasing productivity from planted forests.

### Containment of Genetically Engineered Trees

Genetic engineering (GE) is able to provide solutions for many of the challenges forestry faces to sustainably increase forest production. Improved wood quantity and quality, processability, biotic, and abiotic stress tolerance and herbicide tolerance (Harfouche et al., 2011; Porth and El-Kassaby, 2014; Etchells et al., 2015; Ault et al., 2016; Zhou et al., 2017) are amongst the traits successfully demonstrated. The recent approval by the Brazilian Regulator for GE Eucalyptus that are able to grow 15–20% faster than the best existing clonal lines (Nature Biotechnology News, 2015) seems likely to lead to first large-scale commercial planting of trees.

There remain well documented regulatory and social challenges associated with commercial planting of GE trees (Porth and El-Kassaby, 2014; Strauss et al., 2016). Gene flow from transgenic trees remains a major concern, particularly as forest trees are virtually undomesticated and pollen is able to disseminate over great distances (DiFazio et al., 2004). Seeds also have the potential to spread, either locally or over distances, depending on the species. Transgene containment through the production of trees that are unable to produce fertile reproductive propagules has the ability to mitigate these concerns and prevent, or severely reduce, the flow of genes via sexual reproduction.

### Invasive Tree Species

Increasing attention is being paid to the ecological, economic, and cultural damage caused by invasive tree species that have “escaped” by seed dispersal from planted forests (Breton et al., 2008; Nuñez et al., 2017). Globally, Pinus species are recognized as among the most widespread and influential of all invasive plants (Richardson and Rejmánek, 2004). These escapes, or wildings, are particularly a problem in the Southern Hemisphere where a large percentage of tree plantations are composed of exotic species (Franzese and Raffaele, 2017). South Africa, New Zealand, and Australia, who were early adopters of exotic conifer plantations, have been joined more recently by several South American nations in facing wilding challenges (Simberloff et al., 2010). For example, in New Zealand several exotic conifer species have become established and now occupy ∼1.8 million ha, and are expanding by about 6% per annum (Froude, 2011). Economic and ecological damage resulting from these wildings is challenging the license to operate, with commercially advantageous, but wilding-prone species such as Douglas-fir (*Pseudotsuga menziesii*). The ability to generate trees that are unable to reproduce would allow control programs to focus on the existing populations and give forest owners freedom to operate for new plantings.

### Increased Wood Production and Other Benefits

The ability to either prevent reproduction or limit the development of reproductive propagules is predicted to boost growth and increase wood production in forest trees by redirecting energy and nutrients to increased vegetative growth (Strauss et al., 1995; Luis and José, 2014). Conclusive evidence for such a reproductive cost is lacking but is supported by evidence that in conifers cone production may utilize a significant proportion of the trees energy and assimilates (Cremer, 1992; Sala et al., 2012; Kramer et al., 2014). Unsurprisingly, in conifers, the long-lived female cones are more energy demanding than the generally more transient male cones (Obeso, 2002). These observations suggest that engineered sterility, particularly female sterility is likely to have a positive impact on vegetative growth and wood production. Long-term growth comparisons between sterile and reproductive trees would provide direct evidence for this and allow quantification of growth differences.

Pollen from many trees cause allergenic reactions and symptoms correlate with exposure (Buters et al., 2012). Planted forests can provide a major source of seasonal allergens (D’amato et al., 2007). For example, allergy to sugi (*Cryptomeria japonica*) pollen is reported to effect 26.5% of the Japanese population (Taniguchi, 2018). The ability to prevent or limit pollen production from planted forests would provide relief to allergy suffers and mitigate potential social license to operate challenges.

## Current Understanding of Reproductive Processes in Forest Trees

Both angiosperm (hardwood) and gymnosperm/conifer (softwood) trees are used as plantation species. Although they share broad similarities in their reproductive processes, there are distinct differences between them.

### Angiosperm Trees

Perhaps no other plant development process has been studied more than flowering. For Arabidopsis, the *in planta* functions of a large number of flowering genes as well as their regulatory network context are known and studies in plants such as rice and petunia have revealed broad functional conservation (Pajoro et al., 2014). These include genes that regulate the transition to flowering, floral organ identity as well as pollen and ovule development. Although advances in sequencing have enabled the identification of flowering gene homologs in diverse angiosperm trees, there are few cases where *in planta* functions have been characterized in trees (Brunner et al., 2017; Klocko et al., 2018). This is due to the long non-flowering period that can last years to decades and that for most species, genetic transformation is a formidable hurdle. Trees also differ from herbaceous plants in the prolonged period between the floral transition and anthesis. In tropical species such as Eucalyptus, this occurs in one season, but temperate species exhibit indirect flowering, with flower development initiated in 1 year and completed the following year (Vining et al., 2015; Brunner et al., 2017). Thus, multi-year field trials that require monitoring of large trees and collecting flowers from the upper portion of the tree crown are typically required to demonstrate sterility or delay of flowering.

Selection of candidate genes for genetic containment in trees based on homology to Arabidopsis flowering genes and gene expression might be straightforward, but such conservation does not necessarily translate to the predicted or desired phenotype. Flowering time genes are attractive targets because prevention of flowering is easier to monitor (e.g., no need to demonstrate flowers are sterile) and to prevent resource allocation to reproduction. However, accumulating evidence supports that tree homologs of various flowering time and floral meristem identity genes have roles in both vegetative and reproductive phenology (Bohlenius et al., 2006; Bielenberg et al., 2008; Hoenicka et al., 2008; Mohamed et al., 2010; Hsu et al., 2011; Azeez et al., 2014; Tylewicz et al., 2015; Parmentier-Line and Coleman, 2016). Targeting such genes for manipulation can thus result in undesired vegetative effects, such as delayed bud flush, in addition to predicted effects on flowering or no effect on flowering (Hoenicka et al., 2012). However, promising results have also been achieved, such as the delayed flowering without growth reduction demonstrated by overexpressing the poplar ortholog of the floral repressor *SHORT VEGETATIVE PHASE (SVP)* (Klocko et al., 2018). Manipulation of floral organ identity genes might be less likely to have vegetative effects as these genes may show stronger conservation of reproductive-only function. For example, considerable evidence supports that the *AGAMOUS (AG)* subgroup of MADS-box genes have reproductive functions not only in angiosperms but also in gymnosperms (Dreni and Kater, 2014). However, even in these cases, results can differ from expectation. For example, downregulation of the conserved floral meristem identity gene *LEAFY (LFY)* in a male poplar genotype induced bisexual and female flowers (Klocko et al., 2018). Despite the challenges, the knowledge gained from gene function and sterility studies in trees along with more detailed and extensive genome-wide expression studies in different angiosperm trees will enable more accurate gene selection for manipulation of only-reproductive traits.

### Gymnosperm Trees

Unlike angiosperms, where there is extensive knowledge of the molecular factors involved in the reproduction process, relatively little is known regarding gymnosperms. A number of putative genes have been identified through comparative analyses of orthologous angiosperm genes, tissue-specific expression analysis or genome sequencing. However, several key floral genes including *FD, SQUAMOSA-* (*SQUA*-) or *SEPALLATA-like* (*SEP-like*) seem to be absent (Becker, 2003; Abe et al., 2005; Zahn et al., 2005; Melzer et al., 2010; Karlgren et al., 2011; Jaeger et al., 2013). Initial research was able to detect orthologs to only the B- and C-genes involved in the control of meristem formation and organ identity in the developing cones (Tandre et al., 1995, 1998; Mouradov et al., 1998; Rutledge et al., 1998; Fukui et al., 2001; Sundström and Engström, 2002; Gramzow et al., 2014; Katahata et al., 2014; Uddenberg et al., 2015). More recently, ABCE model prototype transcription factors, genes that define the developmental flower organ model (ABC(DE)) in angiosperms, have been confirmed in gymnosperms (Chen et al., 2017). Conifer-specific genes such as the *DEFICIENS-AGAMOUS-LIKE* (*DAL)* and *NEEDLY* have also been identified but functional knowledge is limited due to the lack of angiosperm orthologs (Carlsbecker et al., 2003, 2004; Rudall et al., 2011). It is not generally possible to predict which of the many vegetative meristems will undergo the reproductive bud transition before changes are initiated making research on reproductive initiation a bold venture (Williams, 2009).

The biggest bottleneck for conifer reproduction research is the inability to carry out functional characterization in an endogenous system which prevents the definitive elucidation of gene function. Testing of gene function in angiosperm model systems has produced inconclusive results. Whilst some such studies have confirmed the function of putative orthologs, others failed to find flowering related differences, found multiple phenotypic alteration or were unable to complement mutants, highlighting the need for a reliable conifer testing system (Rutledge et al., 1998; Tandre et al., 1998; Shindo et al., 2001; Sundström and Engström, 2002; Carlsbecker et al., 2003, 2004; Nilsson et al., 2007; Shiokawa et al., 2008; Klintenas et al., 2012; Katahata et al., 2014; Liu et al., 2018). As discussed above for angiosperm trees, it might be challenging to identify flowering time gene homologs in conifers that do not have roles in vegetative development that make their manipulation for reproductive sterility problematic. For example, the gymnosperm *FLOWERING LOCUS T-like* subfamily has been suggested to have roles in both vegetative and reproductive phenology (Klintenas et al., 2012; Karlgren et al., 2013; Nystedt et al., 2013; Liu et al., 2016). However, the lack of characterization in an endogenous system means that the function of the sub-family members remains unresolved.

## Engineered Sterility in Trees

The increasingly sophisticated understanding of the molecular processes underpinning sexual reproduction described above has facilitated the successful demonstration of a number of sterility strategies in plants. Chief amongst these are strategies using ablation of reproductive cells or structures and the inactivation or suppression of genes essential for normal reproductive processes. Here, we highlight only a selection of sterility approaches and refer readers to Brunner et al. (2007) and Hoenicka et al. (2016b) for additional examples.

The use of cell or tissue-specific promoters to direct the expression of cytotoxic genes (Palmiter et al., 1987) to reproductive tissues has been widely used to investigate and modify reproductive development in plants (Goldman et al., 1994; Beals and Goldberg, 1997). Numerous examples exist of using this technology in plants to generate male and female sterility (Mariani et al., 1990; Goldman et al., 1994; De Block et al., 1997). Complete (dual male and female) sterility has also been achieved using either independent male- and female-specific promoters or a single promoter targeting both tissues simultaneously (Liu and Liu, 2008; Huang et al., 2016). Male sterility using this cell ablation strategy has been demonstrated in both hardwood and softwood trees via expression of the *BARNASE* gene from *Bacillus amyloliquefaciens* under the control of reproductive tissue-specific promoters (Table 1). A key requirement for a cell ablation strategy is a promoter that tightly directs expression of the cytotoxin to the desired reproductive tissue to prevent pleiotropic effects on non-reproductive tissues. The conservation of expression of some floral genes has facilitated the use of a number of well characterized promoters across species (Strauss et al., 1995). Indeed, an anther-specific promoter derived from *Pinus radiata* has been used to express the *BARNASE* gene in both a softwood (pine) and hardwood (Eucalyptus) tree to deliver male sterility (Zhang et al., 2012). We are unware of dual male/female *BARNASE*-mediated sterility being demonstrated in trees without negative pleiotropic effects (Lemmetyinen et al., 2004) but this should be possible if a suitable promoter is used.

**Table 1:** Examples of Engineered Sterility in Forest Trees.

Species	Sterility target	Strategy	Candidate gene(s)	Reference
**Angiosperm trees**				
*Betula pendula*	Male	Cell ablation inflorescences	*BpFULL1::barnase*	Lännenpää et al., 2005
*Eucalyptus occidentalis*	Male	Cell ablation male flowers	*PrMC2::barnaseH102E*	Zhang et al., 2012
*Populus tremula x tremuloides*	Male	Cell ablation tapetum	*TA29:: barnase*	Elorriaga et al., 2014
*Populus alba*	Female	Suppression via RNAi	*PtLFY*	Klocko et al., 2016a
*Populus alba*	Female	Suppression via RNAi	*PtAG*	Lu and Strauss, 2018
*Populus tremula x tremuloides*	Male	Suppression via RNAi	*LFY/AG*	Klocko et al., 2018
**Gymnosperm trees**				
*Pinus rigida x. P. taeda*	Male	Cell ablation male cones	*PrMC2*-*barnaseH102E*	Zhang et al., 2012


RNA interference (RNAi) is a well proven homology-dependent gene silencing technology that involves double-stranded RNA directed against a target gene or its promoter region (Mansoor et al., 2006). Numerous demonstrations of engineered sterility through the suppression of genes essential for normal reproduction are available in angiosperm species (Wang et al., 2012). RNAi silencing has been used in angiosperm trees to engineer sterility with constructs targeting *LFY* and *AG* successfully producing sterile trees (Table 1). The production of male and female sterile plants via the use of chimeric repressors targeting transcription factors involved in flower development has also been demonstrated (Mitsuda et al., 2006; Katahata et al., 2014). In conifers the use of gene suppression methods to prevent reproduction has not been demonstrated even though such methods have been widely used to investigate wood quality traits (Wagner et al., 2005, 2009; Souza et al., 2007; Trontin et al., 2007). Attempts have been reported of expressing conifer flowering-associated genes in an endogenous system (Karlgren et al., 2013) but these studies have not directly sought to address sterility.

This lack of success in conifers reflects both a lack of fundamental knowledge regarding conifer reproduction and the inherent difficulties in working with conifers including the long timescale required for testing. For example, attempts to investigate the effects of over-expressing the Arabidopsis *LFY* gene in *P. radiata* were not informative as neither modified or control plants initiated reproduction during the 8 years that the trees were grown (NZ-EPA, 2008; Lottmann et al., 2010).

## Future Outlook and Challenges

The social, legal and ecological impacts of sterile trees is still controversial (Williams, 2005; Kazana et al., 2015; Strauss et al., 2017). Although sterility provides mitigation for some of the social and ecological objections to the deployment of both GE trees and species with the potential to become invasive, this may be challenged if the sterility technology is itself GE.

The recent development of a number of new breeding technologies, including gene editing, that are already seeing widespread application in crop species (Nekrasov et al., 2017; Waltz, 2018) have great potential in forest trees. Site-directed mutagenesis would allow the inactivation of genes that are essential for normal reproductive processes and the generation of sterile trees. Gene editing-mediated mutagenesis would be particularly advantageous in forest trees where, to date, mutagenesis breeding has played an extremely limited role. The permanent inactivation of a gene would provide assurance of enduring containment and reduce concerns associated with the stability of long-term transgene expression associated with silencing of over-expression technologies (Li et al., 2008). Site directed mutagenesis via CRISPR-cas9 has been demonstrated in a number of tree species including Poplar (Fan et al., 2015) where mutagenesis of genes involved in flowering (Elorriaga et al., 2018) has also been shown. To date, gene editing has not been published in conifer species. However, the existence of a small number of natural spontaneous sterile conifer mutants (Orr-Ewing, 1977; Wilson and Owens, 2003; Rudall et al., 2011) suggest that a targeted-mutagenesis strategy would be successful if suitable targets can be identified.

Although the regulatory landscape regarding gene editing technologies remains complex, it is likely that in many jurisdictions versions of the technology that do not include foreign DNA in the final organisms will not be regulated as GMOs (Waltz, 2016; Davison and Ammann, 2017; Ishii and Araki, 2017). This would provide a more straightforward and less costly route to commercial release than is currently the case for products of GE technology (Waltz, 2018). This regulatory approach would hold particular promise in applications where sterility is a standalone trait, such as for the control of invasive tree species, rather than providing a means of containment for other (GE) traits. This strategy would require DNA-free editing technologies as outcrossing of transgenes would not be possible with sterile trees.

The second major challenge has been the inability to carry out timely prototyping of sterility constructs in commercially important species. To facilitate testing in conifers it is desirable to develop a system analogous to the Poplar model system (Jansson and Douglas, 2007; Douglas, 2017) which has allowed relatively rapid prototyping of sterility constructs (Klocko et al., 2018). Although effective transformation systems exist for a number of commercially important conifers including *P. radiata, P. taeda*, and *Picea abies*, these species have long pre-reproductive juvenile growth periods that limits their use as sterility-testing platforms (Tang and Newton, 2003; Uddenberg et al., 2015). Some conifer species are able to reproduce at a much younger age (Righter, 1939; Pharis et al., 1987; Uddenberg et al., 2013) or can be induced to undergo early reproduction. Such precocious reproduction has been demonstrated in both hardwoods and softwoods by grafting onto older rootstock (Simak, 1978; Zhang et al., 2012), and by the application of external stimuli such as hormone treatments (Pharis et al., 1965; Ross and Pharis, 1985; Meilan, 1997). Stable introduction of *FT* transgenes induced precocious fertile flowers in Eucalyptus (Klocko et al., 2016b) and in Populus when combined with a low temperature treatment (Hoenicka et al., 2016a). In fruit trees, viral vectors that express floral promoters or silence repressors induced early flowering (Velázquez et al., 2016; Yamagishi et al., 2016). Although these offer potential routes to earlier testing of sterility strategies, developing the required tissue culture and transformation capabilities for a new tree species remains a significant barrier.

The increasing availability of genome and transcriptome resources for forest trees is providing new insights into reproductive processes. This is reducing the reliance on non-tree model systems and providing novel species-specific knowledge of reproductive processes and candidate genes for modification. The development of gene-editing-based targeted mutagenesis is likely to be the most attractive route to engineered sterility as it offers precise and predictable modifications combined with assurance of phenotypic stability. The lack of global consensus on the regulation of gene editing technology remains a barrier to research investment and commercialization and complicates the public debate that must go hand-in-hand with progress toward implementation.

## Author Contributions

All authors listed have made a substantial, direct and intellectual contribution to the work, and approved it for publication.

## Conflict of Interest Statement

The authors declare that the research was conducted in the absence of any commercial or financial relationships that could be construed as a potential conflict of interest.
